# Optimization of the Hydrolysis of Safflower Oil for the Production of Linoleic Acid, Used as Flavor Precursor

**DOI:** 10.1155/2015/594238

**Published:** 2015-06-04

**Authors:** Marya Aziz, Florence Husson, Selim Kermasha

**Affiliations:** ^1^Department of Food Science and Agricultural Chemistry, McGill University, 21111 Lakeshore, Sainte-Anne-de-Bellevue, QC, Canada H9X 3V9; ^2^AgroSup Dijon, Université de Bourgogne, 1 Esplanade Erasme, 21000 Dijon, France

## Abstract

Commercial lipases, from porcine pancreas (PPL), *Candida rugosa* (CRL), and *Thermomyces lanuginosus* (Lipozyme TL IM), were investigated in terms of their efficiency for the hydrolysis of safflower oil (SO) for the liberation of free linoleic acid (LA), used as a flavor precursor. Although PPL, under the optimized conditions, showed a high degree of hydrolysis (91.6%), its low tolerance towards higher substrate concentrations could limit its use for SO hydrolysis. In comparison to the other investigated lipases, Lipozyme TL IM required higher amount of enzyme and an additional 3 h of reaction time to achieve its maximum degree of SO hydrolysis (90.2%). On the basis of the experimental findings, CRL was selected as the most appropriate biocatalyst, with 84.1% degree of hydrolysis. The chromatographic analyses showed that the CRL-hydrolyzed SO is composed mainly of free LA.

## 1. Introduction

Polyunsaturated fatty acids (PUFAs), such as linoleic acid (LA,* cis,cis-*9,12-octadecadienoic acid), are considered as being the precursors for the synthesis of flavors, mainly lipid-derived aldehydes and alcohols [1]. Although pure commercial LA has been extensively used as a model substrate in research for the synthesis of lipid-derived flavors, its high cost limited its use at the industrial level [1, 2]. Hence, edible oils could be alternative and economical sources for LA, the PUFA of interest. Among the most common commercial edible oils, safflower oil (SO) is characterized by its high content (70 to 80%) of LA [3], its availability, and its low cost [2]. The literature [1, 4, 5] suggested the necessity of hydrolyzing the edible oils, prior to their use as sources of LA for the production of flavors. Although the use of hydrolyzed edible oils, including sunflower [1] and safflower [4], for the synthesis of hexanal has already been reported in literature, little information was available on the nature of the hydrolysis process. The harsh conditions of high pressure and temperature of the conventional process of hydrolysis of edible oils could lead to the polymerization of fats, resulting in extremely dark free fatty acids (FFAs) that would limit its industrial use [6, 7]. The use of enzymes for the hydrolysis of oils, with a high content of PUFAs, could be hence of great interest since enzyme-catalyzed hydrolytic reactions offer simple operational procedure, high fatty acid selectivity, and low cost as compared to their chemical counterparts [2, 7].

Lipases (triacylglycerol (TAG) hydrolases, EC 3.1.1.3) are by far the most commonly used enzymes for the hydrolysis of oils [6], resulting into FFAs, glycerols, and acylglycerols [3]. Because of its ability to efficiently hydrolyze edible oils, the lipase from* Candida rugosa* (CRL) has been extensively reported in literature [2, 7, 8]. Moreover, Freitas et al. [2] showed that the hydrolyzed soybean oil, obtained by lipase from porcine pancreas (PPL), has the highest LA content among the investigated enzymes. Although commercial PPL and CRL have been commonly reported in literature [2, 8] for their use in the hydrolysis of oils, the lipase from* Thermomyces lanuginosus* (Lipozyme TL IM) is rather widely used for the synthesis of structured lipids [9]; however, its immobilization on granular silica carrier makes it an attractive lipase, since it facilitates its dispersion, recovery, and reusability as well as providing better stability as compared to its free counterpart [10].

The overall objective of this study was to determine the capacity of selected commercial lipases to hydrolyze SO for the liberation of free LA for the application as a flavor precursor. The specific objectives were to investigate the effects of selected parameters on the degree of hydrolysis of SO and to determine the composition of the hydrolyzed SO in free fatty acids as well as mono-, di-, and triacylglycerols.

## 2. Materials and Methods

### 2.1. Materials

Edible safflower oil (SO) was purchased from a local market; its composition in fatty acids (%) was determined by gas chromatography (GC) to be 7.5, 3.8, 16.5, and 72.2 of C_16:0_, C_18:0_, C_18:1_, and C_18:2_, respectively. Two non-immobilized lipases from Candida rugosa type VII (CRL) and from porcine pancreas type II (PPL) as well as sodium methoxide (CH_3_ONa) were purchased from Sigma Chemical Co. (St. Louis, MO). The hydrolytic activity for CRL was 1,449 unit/mg solid enzyme, whereas that for PPL was 59.45 unit/mg solid enzyme when olive oil is used at pH 7.7. In addition, an immobilized lipase from* Thermomyces lanuginosus* (Lipozyme TL IM), with 20 unit/mg solid enzyme, was obtained from Novo Nordisk (Copenhagen, Denmark). Both PPL and Lipozyme TL IM are 1,3-regiospecific lipases, whereas CRL is a nonspecific lipase. Polyoxyethylene sorbitan monolaurate (Tween-20), sodium hydroxide (NaOH), Sigma 7-9, Whatman 1PS filter, and organic solvents of high-performance liquid chromatography (HPLC) grade were purchased from Fisher Scientific (Fair Lawn, NJ). 12 N Hydrochloric acid (HCl), 12 N sulfuric acid (H_2_SO_4_), and 1 N standard HCl solution were also purchased from Fisher Scientific. Fatty acid methyl ester (FAME), thin-layer chromatography (TLC), and HPLC standards were purchased from Nu-Chek Prep (Elysian, MN).

### 2.2. Hydrolytic Reaction Preparation

The hydrolytic reaction was carried out in 125 mL Erlenmeyer flasks, incubated at 45°C, with continuous shaking at 250 rpm, using an orbital shaker-incubator (New Brunswick Scientific Co., Inc., Edison, NJ). The final SO concentration (6 mM) in the enzymatic reaction medium was calculated on the basis of its content in trilinolein (TLA), as determined by GC. The reaction mixture consisted also of 0.5% (v/v) Tween-20 and sufficient Tris-HCl buffer solution (0.1 M) to adjust the final volume of the mixture to 30 mL. The pH for CRL, PPL, and Lipozyme TL IM was 7.2, 7.7, and 8.0, respectively. The enzymatic reaction was initiated by the addition of 1 mL of either CRL or PPL suspension (100 mg solid enzyme/mL), prepared in 0.1 M Tris-HCl buffer solution at pH 7.2 and pH 7.7, respectively, or by a direct addition of 100 mg of granular Lipozyme TL IM to the reaction mixture. In the case of CRL, the enzyme suspension was homogenized for 10 sec, prior to its addition to the reaction mixture, using the Sonicator Ultrasonic Processor (Model XL2020, Heat Systems, Inc., Farmingdale, NY). Blank samples, containing all components except the enzyme preparation, were carried out in tandem with the enzymatic trials.

### 2.3. Determination of the Degree of Hydrolysis of SO

At specific intervals, flasks were removed from the orbital shaker-incubator and the degree of hydrolysis was determined by titration, using Mettler-Toledo DL58 automated titrator (Mettler-Toledo, Schwerzenbach, Switzerland) equipped with a 10 mL burette and a sample changer ST20A. The system was computer-controlled by LabX Software. The equivalent point was determined by measuring the pH change, with the use of a DG101-SC pH electrode, which was calibrated prior to its use. Standard HCl solutions of 0.1 and 0.2 N were used to standardize 0.1 and 0.2 N NaOH solutions, respectively. A standardized solution of 0.1 N NaOH was used for the titration, when the pH of the reaction mixture was greater than 6.7; however, a 0.2 N NaOH standardized solution was used at pH lower than or equal to 6.7. In order to meet the defined application, preliminary tests were conducted to adjust the equivalent point method 90005 (LabX Software).

The degree of hydrolysis was defined as the percentage weight of FFAs in the sample divided by the initial weight of SO sample and calculated according to the following equation:

(1) Oil  hydrolysis%=Vte−Vto×M×MwWSO×100,

where *V*_to_ and *V*_te_ were the volumes of NaOH solution used, respectively, for the sample at time 0 and at the equivalent point; [*M*] is the molar concentration of NaOH solution used (0.1 N or 0.2 N); *M*_*w*_ is the molecular weight of linoleic acid (LA) (280.45 g/mol); *W*_SO_ is the initial weight of the SO sample.

### 2.4. Effect of Reaction Time on the Hydrolysis of SO

The effect of reaction time, 0 to 8 h, on the hydrolysis of SO was investigated, using CRL, Lipozyme TL IM, and PPL as biocatalysts.

### 2.5. Effect of Enzyme Concentration on the Hydrolysis of SO

The effect of enzyme concentration, 0.0 to 4.0 mg solid enzyme/mL reaction mixture, on the hydrolysis of SO was studied, at constant reaction time of 3 h for CRL and PPL and 6 h for Lipozyme TL IM.

### 2.6. Effect of Substrate Concentration on the Hydrolysis of SO

Using the selected lipases, the degree of hydrolysis of SO was investigated over a wide range of substrate concentrations, 0 to 8 mM. The enzymatic reaction was carried out for 3 h for CRL and PPL and 6 h for Lipozyme TL IM, using the optimal biocatalyst amount of 1.3, 2.0, and 3.3 mg solid enzyme/mL reaction mixture for CRL, PPL, and Lipozyme TL IM, respectively.

### 2.7. Effect of Temperature on the Hydrolysis of SO

In order to favor the hydrolysis of SO, the effect of temperature, 30 to 55°C, on the hydrolytic reaction, catalyzed by the investigated biocatalysts, was investigated. The optimized conditions used for CRL and PPL were 1.3 and 2.0 mg solid enzyme/mL reaction mixture, with 6 and 1 mM of SO, respectively, and 3 h of reaction time. For Lipozyme TL IM, 3.3 mg of granular enzyme/mL reaction mixture, 7 mM of SO, and 6 h of reaction time were used.

### 2.8. Effect of pH on the Hydrolysis of SO

Under the optimized reaction conditions, described previously, the effect of pH on the hydrolysis of SO by the selected lipases was investigated, using different buffer solutions (0.1 M) of potassium phosphate (pH 6.5 to 6.7) and Tris-HCl (pH 7.0 to 8.7).

### 2.9. Recovery of Hydrolyzed SO

After hydrolysis, the flasks were withdrawn from the orbital shaker-incubator and the enzymatic reaction was halted by the addition of drops of 1 N HCl solution (pH 3.4). The hydrolyzed SO (HSO) was extracted 3 times with hexane (1 : 1, v/v), with rigorous agitation for 5 min; the reaction mixture was then left for decantation for 10 min. All traces of protein, water, and Tween-20 were eliminated by suction filtration, using a Whatman 1PS filter paper. The hexane was evaporated, using a SpeedVac System (Model AES2010, Thermo Savant, Holbrook, NY). Any residual traces of hexane were removed by a gentle stream of nitrogen and the recovered HSO was stored at −80°C for further analyses.

### 2.10. Determination of the Oxidation Content of the HSO

The recovered HSO was subjected to high-performance liquid chromatography (HPLC) analysis, according to the procedure developed in our laboratory [11]. The HPLC system was a Beckman System (Beckman Instruments Inc., San Ramon, CA), equipped with a computerized integration and data handling system (Model 126), using a 32 Karat Software version 8.0 and a UV-VIS diode-array detector (DAD-Model 168). The recovered HSO was separated on a normal-phase- (NP-) ZORBAX Rx-SIL column (250 × 4.6 mm, i.d. 5 *μ*m, Alltech Associates Inc., Deerfield, IL). The absorbance was measured simultaneously at 205 and 234 nm to monitor the presence of any linoleic acid hydroperoxides (HPODs) in the recovered HSO, resulting from the LA autooxidation. The isocratic eluent system was a mixture of hexane/2-propanol/acetic acid (990 : 10 : 1, v/v/v) at a flow rate of 1.0 mL/min, for a total elution time of 26 min.

### 2.11. Characterization of End Products of SO Hydrolysis by CRL

#### 2.11.1. TLC Analysis of HSO

Aliquots of SO and HSO, obtained by CRL, were analyzed by thin-layer chromatography (TLC) on silica gel 60 plates, with fluorescent indicators (Whatman, Fisher Scientific). The TLC was carried out with a saturated solvent mixture of hexane/diethyl ether/acetic acid (140 : 60 : 1, v/v/v). The TLC plates were visualized under visible light, after their spraying with 20% (v/v) sulfuric acid. Acylglycerols standard (AGs-STD), composed of 25% equal amount of methyl linoleate, mono-, di-, and trilinolein, as well as a mixture of free fatty acid standards (LA/OA-STD), containing 67% linoleic acid and 33% oleic acid, was used as references. Retention factor (*R*_*f*_), defined as the migration distance of a component over that of the solvent, was used to characterize the different components of SO and HSO.

#### 2.11.2. HPLC Analysis of HSO

The high-performance liquid chromatography (HPLC) analysis of HSO, obtained by CRL, was carried out according to the method developed in our laboratory [12]. The separation of different components was performed on an Agilent ZORBAX SB-C18 reversed-phase column (250 × 4.6 mm, i.d. 5 *μ*m), using the Beckman HPLC system. A volume of 20 *μ*L sample was diluted to 320 *μ*L with isopropanol. The diluted sample was filtered and 20 *μ*L of the filtrate was subjected to HPLC analysis. The elution of the injected sample was carried out by a gradient solvent system, using methanol as solvent (A) and isopropanol as solvent (B). The elution was initiated by an isocratic flow of 100% of solvent A for 10 min, followed by a 10 min linear gradient to 40 and 60% of solvents A and B, respectively, and then to 100% of solvent B for 10 min period. The elution was maintained for an additional period of 5 min before reverting it to the initial conditions (100% solvent A), followed with an equilibration period of 10 min for the next sample. The flow was 1 mL/min and the detection was performed at 215 nm for monitoring the lipid components. SO was also analyzed by HPLC to take into account the presence of any trace amounts of FFAs, mono-, and diacylglycerols.

#### 2.11.3. Preparation of Fatty Acid Methyl Esters

Fatty acid methyl esters (FAMEs) of SO and HSO were prepared according to Badings and De Jong [13] to determine their composition in FA and FFA, respectively. For the preparation of FAMEs of SO, fifty mg of SO was diluted in 0.6 mL of hexane, followed by the addition of 60 *μ*L of 2 M sodium methoxide in 20% methanol. The mixture was incubated in reciprocal shaking water-bath (Model 25, Precision Scientific, Chicago, IL) at 65°C. After 20 min of incubation, 1 mL of 10% sulfuric acid solution prepared in absolute methanol was added, followed by its incubation at 85°C for 30 min. For the preparation of the free FAMEs of HSO, fifty mg of the recovered HSO was diluted in 0.6 mL of hexane, followed by the addition of 1 mL of 20% HCl solution prepared in absolute methanol. The mixture was incubated in reciprocal shaking water-bath at 85°C for 15 min. The FAMEs of SO and HSO were then extracted 3 times with 4 mL hexane. The mixture was centrifuged (1,600 rpm, 5 min) and the upper layer was recovered, where the organic solvent was evaporated with the use of a SpeedVac System (Model AES1010, Savant, Holbrook, NY). The residual traces of the organic solvent were removed by a gentle stream of nitrogen. The recovered FAMEs were diluted, with hexane, prior to gas chromatography (GC) analysis.

#### 2.11.4. GC Analysis of FAMEs

The fatty acid composition of SO as well as the free ones of the HSO, obtained by CRL, was analyzed, by gas-liquid chromatography (GC), using Agilent 6890 Series (GC) system (Agilent Technologies, Wilmington, NC), equipped with computerized integration and data handling (GC ChemStation G2075AA, version A.09.03, Agilent) software and a flame ionization detector (FID). Samples of 1 *μ*L were injected, using the split mode injection. The separation of the different FAMEs was performed on a HP-INNOWax polyethylene glycol fused capillary column (30 m × 0.25 mm, i.d. 0.25 *μ*m film thickness, Agilent), with a flow rate of carrier gas (He) of 1 mL/min. The oven temperature was programmed as follows: 150°C during the first 1 min and then it increased to 200°C, at 10°C/min; the temperature was then increased to 220°C, at 1°C/min, and it was held for 5 min before a final increase to 225°C, at 1°C/min, where it was held for 5 min. The flow rates for the hydrogen and air were set at 40 and 400 mL/min, respectively. The split injector and FID temperatures were set at 220 and 230°C, respectively.

### 2.12. Statistical Analyses

All enzymatic assays were performed in triplicate. The relative standard deviation (RSD) was calculated as the standard deviation (SD) of triplicate trials divided by their mean multiplied by 100. All statistical analyses were performed, using SigmaPlot for Windows version 11. One-way analysis of variance (ANOVA) test was used to determine the differences among several groups, followed by the Holm-Sidak test for pairwise comparisons. For one-way ANOVA, a difference was considered significant at *P* < 0.05.

## 3. Results and Discussion

### 3.1. Effect of Reaction Time on the Enzymatic Hydrolysis of SO

**Figure 1:**
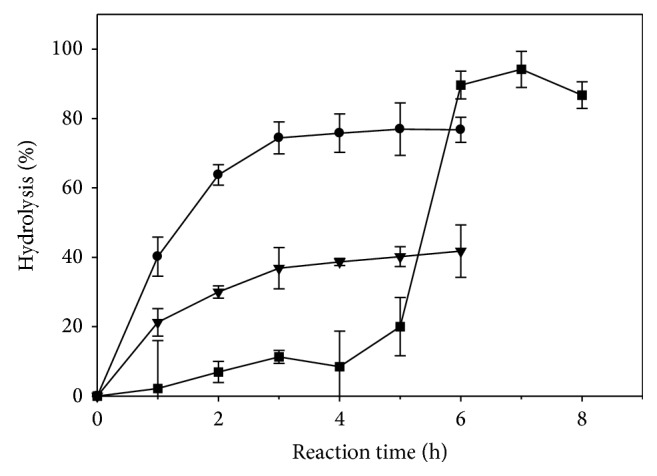
Effect of reaction time on the hydrolysis of 6 mM safflower oil, using lipases from* Candida rugosa* type VII (CRL) (●), porcine pancreas type II (PPL) (▼), and* Thermomyces lanuginosus* (Lipozyme TL IM) (■). The biocatalyst amount was 3.3 mg of solid enzyme/mL reaction mixture for CRL and PPL and 3.3 mg of granular enzyme/mL reaction mixture for Lipozyme TL IM.

Using the selected lipases, the time course for the enzymatic hydrolysis of safflower oil (SO) is shown in Figure 1. Over a wide range of reaction time (0 to 6 h), the use of PPL as biocatalyst showed that the level of hydrolysis of SO after 3 and 6 h was 36.9 and 41.8%, respectively. With the use of CRL as biocatalyst, the degree of hydrolysis of SO significantly (*P* < 0.05) increased from 0.0 to 74.4%, within 3 h of reaction; however, a 2.3% increase in the degree of hydrolysis after an additional 3 h of reaction was insignificant (*P* > 0.05). On the other hand, the immobilized Lipozyme TL IM-catalyzed reaction showed a significant (*P* < 0.05) increase in the degree of hydrolysis, from 0.0 to 20.0%, within the first 5 h of reaction, followed by a dramatic significant (*P* < 0.05) increase in the degree of hydrolysis to 89.6% after one additional hour. However, there was an insignificant (*P* > 0.05) decrease in the degree of hydrolysis after an additional 2 h of reaction time reaching 86.7%. Pairwise comparisons of the degrees of hydrolysis of SO, obtained at 3 h for PPL and CRL and 6 h for Lipozyme TL IM, were found to be statistically significant (*P* < 0.05). The difference in the hydrolytic efficiency of the investigated lipases could be due to the differences in their affinity and regioselectivity towards the TAGs of the SO.

The experimental findings (Figure 1) for PPL and CRL are in agreement with those of Freitas et al. [2], who indicated that microbial lipases, mainly CRL and Lipolase from* Thermomyces lanuginosus*, were more effective than those from animal sources, such as PPL, in their hydrolysis of soybean oil; there was a 70.0 and 53.0% hydrolysis after 24 h of reaction for CRL and Lipolase as opposed to 23.0% for PPL as biocatalyst. Wu et al. [14] also showed that microbial lipases, from* C. rugosa*,* Chromobacterium viscosum*,* Rhizomucor miehei*, and* Rhizopus* sp., exhibited higher hydrolytic activity towards olive oil as compared to those from animal sources. The higher degree of hydrolysis, obtained with CRL (Figure 1), could be associated with its ability to liberate all types of acyl chains [2, 15]. Freitas et al. [2] suggested that the lower degree of hydrolysis, obtained by PPL, may be due to the presence in the commercial preparation of other enzymes, such as cholesterol esterase, carboxypeptidase *b*, *α*-chymotrypsin, and other unknown hydrolyses.

In terms of the reaction time, Wu et al. [14] showed that CRL exhibited 57-time higher hydrolytic activity, obtained after only 30 min of reaction, than that of PPL. Serri et al. [7] indicated that the highest degree of hydrolysis of palm oil by CRL was obtained after 90 min of enzymatic reaction. Similar findings were reported by Noor et al. [6] upon the hydrolysis of palm oil by lipase SP398, from* Humicola lanuginosa*. Fu et al. [16] reported that the optimum reaction time for the hydrolysis of soybean oil, lard, and coconut oil by lipase 8901, from* Aspergillus *sp., was 1, 3, and 5 h, respectively; these authors suggested that the variation in the reaction time could be due to the differences in the TAG composition of the investigated oils. Based on the experimental findings, further studies were performed, using the reaction time of 3 h for PPL and CRL and 6 h for Lipozyme TL IM.

### 3.2. Effect of Enzyme Concentration on the Enzymatic Hydrolysis of SO

**Figure 2:**
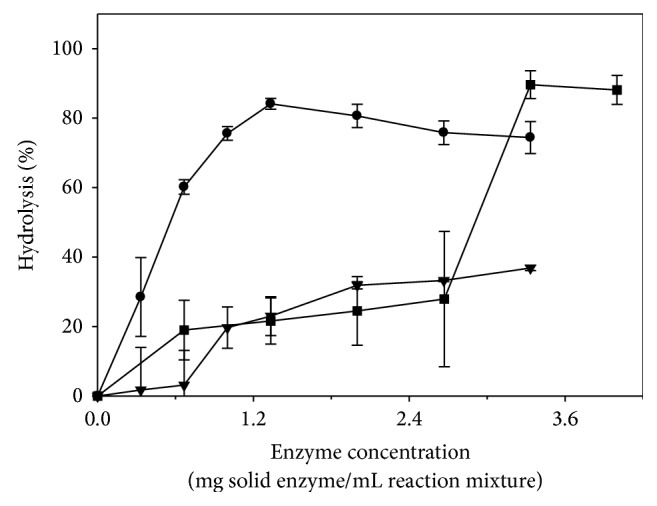
Effect of enzyme concentration, using lipases from* Candida rugosa* type VII (CRL) (●), porcine pancreas type II (PPL) (▼), and* Thermomyces lanuginosus* (Lipozyme TL IM) (■), on the hydrolysis of 6 mM safflower oil.

Figure 2 shows the effect of enzyme concentration on the hydrolysis of SO by the investigated lipases. Using CRL (1.3 mg solid enzyme/mL reaction mixture) as biocatalyst, the highest degree of hydrolysis of 84.1% was obtained; however, there was an insignificant (*P* > 0.05) change in the degree of hydrolysis when the amount of enzyme was increased to 3.3 mg solid enzyme/mL reaction mixture. The results also indicated that the degree of hydrolysis of SO by PPL, with an enzyme concentration lower than 0.7 mg solid enzyme/mL reaction mixture, was deviated from the hyperbolic Michaelis-Menten plot; however, the degree of hydrolysis was significantly (*P* < 0.05) increased from 0.0 to 31.9%, when 2.0 mg solid enzyme/mL reaction mixture was used, followed by an insignificant (*P* > 0.05) increase to 36.9%, with 3.3 mg solid enzyme/mL reaction mixture. Using Lipozyme TL IM as biocatalyst, a limited but significant (*P* < 0.05) increase, from 19.0 to 27.9%, in the degree of hydrolysis was obtained when the amount of enzyme was increased from 0.7 to 2.7 mg granule/mL reaction mixture, followed by a significant (*P* < 0.05) dramatic increase to a maximum of 89.6%, with the use of 3.3 mg granule/mL reaction mixture; however, an increase in the amount of enzyme to 4.0 mg granule/mL reaction mixture did not result in a significant (*P* > 0.05) change in the degree of hydrolysis. Although the use of Lipozyme TL IM resulted in the highest degree of hydrolysis of SO, it required 1.7- and 2.5-time amount of enzyme as compared to that of PPL and CRL, respectively. Moreover, while there was a statistical significant (*P* < 0.05) difference in the degree of hydrolysis of SO among CRL, PPL, and Lipozyme TL IM at 1.3, 2.0, and 3.3 mg solid enzyme/mL reaction mixture, respectively, there was an insignificant statistical (*P* > 0.05) difference in the degree of hydrolysis of SO between CRL and TL IM at 1.3 and 3.3 mg solid enzyme/mL reaction mixture, respectively.

The literature [7, 17, 18] reported similar findings for the hydrolysis of various vegetable oils by CRL. The limited increase in the degree of hydrolysis at higher enzyme concentrations may be due to the enzyme-saturation at the interface between the oil and the aqueous phase in which any further increase in the amount of enzyme would show negligible effect [7, 18, 19]. O'Connor and Bailey [20] indicated that the hydrolysis of tributyrin by PPL exhibited a sigmoidal curve, which was attributed to cooperative interactions between the lipase and its coenzyme, present in its preparation; these authors suggested that PPL could be allosterically regulated by effectors. On the basis of these experimental findings, the optimum amount of CRL, PPL, and Lipozyme TL IM for the hydrolysis of SO was determined to be 1.3, 2.0, and 3.3 mg solid enzyme/mL reaction mixture, respectively, and was used consequently throughout this study.

### 3.3. Effect of Substrate Concentration on the Enzymatic Hydrolysis of SO

**Figure 3:**
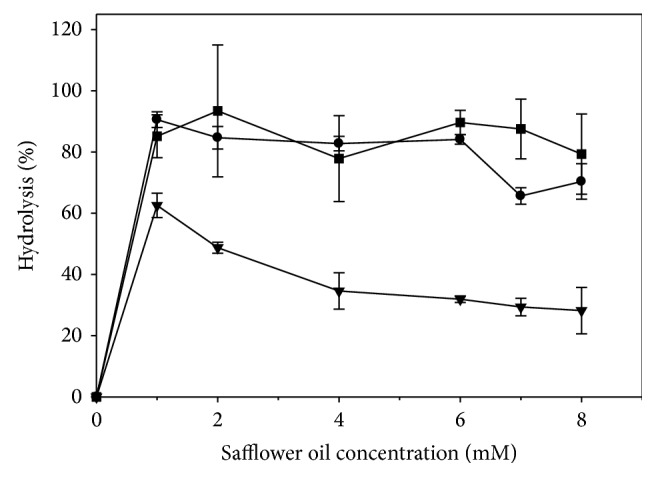
Effect of substrate concentration on the hydrolysis of safflower oil, using lipases from* Candida rugosa* type VII (CRL) (●), porcine pancreas type II (PPL) (▼), and* Thermomyces lanuginosus* (Lipozyme TL IM) (■). The optimal biocatalyst amount was 1.3 and 2.0 mg solid enzyme/mL reaction mixture for CRL and PPL, respectively, and 3.3 mg granular enzyme/mL reaction mixture for Lipozyme TL IM.

Using the investigated lipases, the degree of hydrolysis of SO was determined over a wide range of substrate concentrations, 0 to 8 mM. The experimental findings (Figure 3) suggest that the lipolytic activity of the investigated lipases deviated from the hyperbolic Michaelis-Menten plot. Using PPL as biocatalyst, the degree of hydrolysis significantly (*P* < 0.05) decreased as a function of substrate concentration, with a maximum of 62.5%, obtained with the use of 1 mM of SO. Using CRL as biocatalyst, there was an insignificant (*P* > 0.05) decrease in the degree of hydrolysis of SO from 90.6 to 84.1% with the increase in substrate concentration, from 1 to 6 mM, before a significant (*P* < 0.05) decrease in the degree of hydrolysis to 70.3%, with the use of 8 mM of SO. Using Lipozyme TL IM as biocatalyst, there was an insignificant (*P* > 0.05) difference in the degree of hydrolysis of SO with the increase in substrate concentration from 1 to 8 mM. In addition, there was an insignificant (*P* > 0.05) difference in the degree of hydrolysis between that for CRL, with 6 mM of SO, and that for PPL and TL IM, using 1 and 7 mM of SO, respectively.

Serri et al. [7] reported that the degree of hydrolysis of palm oil by CRL decreased steadily with the increase in substrate concentration; it was suggested that such behavior may be due to the saturation of the active site of lipase by the oil phase, limiting hence the availability of the enzyme for the substrate. Kwon and Rhee [15] reported similar findings for the free and immobilized CRLs, with tributyrin concentration greater than 3 and 4% (v/v), respectively; this was attributed to an increase in the hydrophobicity of the system, reducing hence the activity of the lipase. O'Connor and Bailey [20] proposed that the enzyme inhibition at higher substrate concentrations may be due to an interaction between the insoluble substrate, such as tributyrin, and the lipase. In contrast, Noor et al. [6] reported that the degree of hydrolysis of palm oil by lipase SP398, from* H. lanuginosa*, increased linearly as a function of oil concentration. Chew et al. [21] indicated similar findings, upon the hydrolysis of palm olein by Lipozyme TL IM. Based on the experimental findings, the optimum substrate concentration for CRL, PPL, and Lipozyme TL IM was determined to be 6, 1, and 7 mM, respectively, and was used for further optimization of SO hydrolysis.

### 3.4. Effect of Temperature on the Enzymatic Hydrolysis of SO

**Figure 4:**
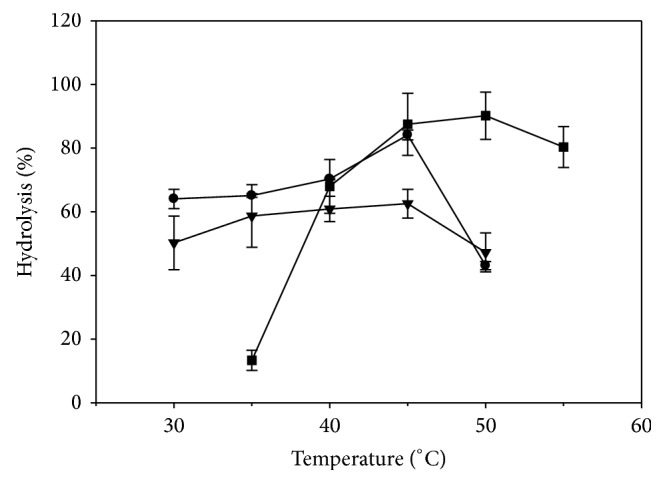
Effect of temperature on the hydrolysis of safflower oil, using lipases from* Candida rugosa* type VII (CRL) (●), porcine pancreas type II (PPL) (▼), and* Thermomyces lanuginosus* (Lipozyme TL IM) (■). The optimal biocatalyst amount was 1.3 and 2.0 mg solid enzyme/mL reaction mixture for CRL and PPL, respectively, and 3.3 mg granular enzyme/mL reaction mixture for Lipozyme TL IM.

The effect of temperature on the enzymatic hydrolysis of SO was investigated. Using CRL as biocatalyst, the results (Figure 4) show a statistical significant (*P* < 0.05) increase in the degree of hydrolysis of SO from 64.1% at 30°C to a maximum of 84.1% at 45°C, before a significant (*P* < 0.05) decrease to 43.1% at 50°C. PPL showed an insignificant (*P* > 0.05) increase in the degree of hydrolysis of SO from 30 to 45°C, followed by a significant (*P* < 0.05) decrease to 47.2% at 50°C. Lipozyme TL IM exhibited a significant (*P* < 0.05) increase in the degree of hydrolysis from 13.3% at 35°C to a maximum of 90.2% at 50°C, followed by an insignificant (*P* > 0.05) decrease to 80.4% at 55°C. The experimental findings could be explained by the fact that the immobilization of the enzyme may have restricted its movement by its contact with the support, enhancing hence its rigidity; as a result, the stability of the enzyme is enhanced with respect to extreme temperatures [22]. While there was a statistical significant (*P* < 0.05) difference in the degree of hydrolysis of SO among PPL, CRL, and Lipozyme TL IM at 35, 45, and 50°C, respectively, there was an insignificant statistical (*P* > 0.05) difference in the degree of hydrolysis between CRL and TL IM at 45 and 50°C, respectively.

The experimental findings for CRL are in agreement with those of Serri et al. [7], who reported that the optimum temperature for the hydrolysis of palm oil was 45°C; these authors suggested that the significant decrease in hydrolysis at temperatures higher than 45°C could be due to the disruption of the enzyme tertiary structure, which could result in its denaturation. Similar findings were also reported by Fadiloğlu and Söylemez [17], when olive oil was hydrolyzed by celite-immobilized CRL. In contrast, Maidina et al. [3] indicated that the hydrolysis yield of SO by CRL at 45°C was less than half of that obtained at 30°C; these authors concluded that the temperature was a crucial parameter for CRL, affecting not only the enzyme activity and its stability but also the state of the reaction medium and/or interface. The differences in the optimal temperature for CRL, obtained by Maidina et al. [3] and those reported in Figure 4, could be due to the choice of the surfactant used for the stabilization of the interface. In addition, Kwon and Rhee [15] investigated the temperature effect on the hydrolysis of tributyrin and triolein, using free and immobilized CRL, where the optimum temperature for the free and immobilized enzyme was 50 and 60°C, respectively. Goswami et al. [8] reported that the optimum temperature for the hydrolysis of castor oil by CRL was 35°C. These authors [8] suggested that the temperature change affected the rate of the lipase-catalyzed hydrolysis of vegetable oils and the thermal inactivation of the enzyme itself, indicating that, at low temperatures, the rate of thermal inactivation of lipase was negligible, whereas that of the hydrolysis of oil increased as a function of temperature increase; however, at high temperatures the rate of thermal deactivation of lipase became more prominent and hence there was a decrease in the rate of the hydrolysis of oil. Based on the experimental findings, further studies were performed for CRL, PPL, and Lipozyme TL IM, using 45, 35, and 50°C as their optimum temperature, respectively.

### 3.5. Effect of pH on the Enzymatic Hydrolysis of SO

**Figure 5:**
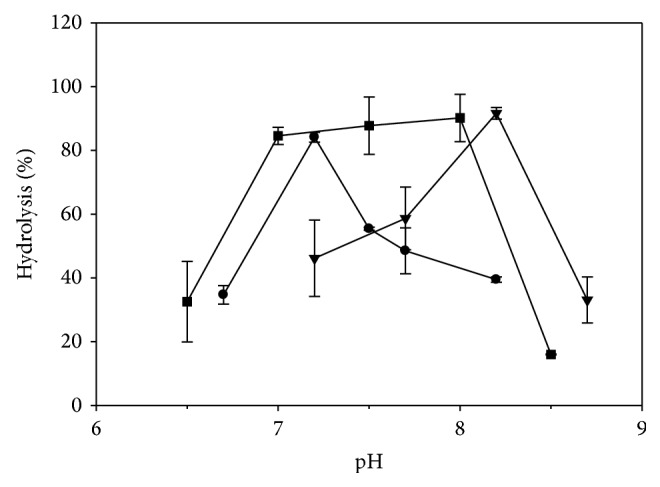
Effect of pH on the hydrolysis of safflower oil, using lipases from* Candida rugosa *type VII (CRL) (●), porcine pancreas type II (PPL) (▼), and* Thermomyces lanuginosus* (Lipozyme TL IM) (■). The optimal biocatalyst amount was 1.3 and 2.0 mg solid enzyme/mL reaction mixture for CRL and PPL, respectively, and 3.3 mg granular enzyme/mL reaction mixture for Lipozyme TL IM.

A change in pH is known to have an effect on the ionization of both free substrate and enzyme [8]. In order to maximize the hydrolysis of SO by the investigated lipases, the enzymatic trials were carried out in a wide range of pH, varying from 6.5 to 8.7. The results (Figure 5) show that CRL exhibited its highest degree of hydrolysis (84.1%) at pH 7.2, whereas PPL reached its maximum (91.6%) at pH of 8.2; however, further increase or decrease in the pH resulted in an overall significant (*P* < 0.05) decrease in the degree of hydrolysis. The degree of hydrolysis of SO by Lipozyme TL IM significantly (*P* < 0.05) increased, from 32.5 to 84.6%, when the pH was changed slightly from 6.5 to 7.0, respectively, followed by an insignificant (*P* > 0.05) increase to 90.2% at pH 8.0; nevertheless, at pH greater than 8.0, the degree of hydrolysis significantly (*P* < 0.05) decreased to 16.0%. The overall experimental findings indicated that the investigated lipases were sensitive to the change in pH value, and this could be attributed not only to the enzyme denaturation but also to the breakdown of the substrate [8]. Serri et al. [7] suggested that the pH could modify the ionization state of the enzyme and, as a result, the activity and selectivity of the enzyme may be altered. The results obtained for CRL (Figure 5) are in agreement with those of Fadiloğlu and Söylemez [17], who reported that the optimum pH value for the hydrolysis of olive oil by the nonimmobilized CRL was determined to be 7.0. Likewise, the hydrolysis of triolein [15] and soybean oil [2] was shown at its maximum at pH 7.0. In addition, Serri et al. [7] showed that the optimum pH for the hydrolysis of palm oil by CRL was 7.5. The results for PPL (Figure 5) are in agreement with those reported by O'Connor and Bailey [20], who used pH 8.0 for the hydrolysis of emulsified tributyrin. In contrast, the PPL-catalyzed hydrolysis of soybean oil was carried out at pH 7.5, resulting in 23.0% hydrolysis [2].

### 3.6. Selection of the Appropriate Biocatalyst

Although the use of PPL, under the optimized conditions, showed (Figure 5) a high degree of hydrolysis (91.6%), its low tolerance towards higher substrate concentrations could limit its use for the hydrolysis of SO. In comparison to CRL, Lipozyme TL IM (Figure 5) required 2.5-time more amount of enzyme and an additional 3 h of reaction time to achieve its maximal degree of hydrolysis of 90.2%. Moreover, the HPLC analysis of the hydrolyzed SO by Lipozyme TL IM showed trace amounts of linoleic acid hydroperoxide isomers (HPODs), suggesting hence that some of the released linoleic acid (LA) had undergone an autooxidation; this oxidation could be due to the slight increase in the optimum temperature of 50°C for Lipozyme TL IM as opposed to that of 45°C for CRL. Although the use of Lipozyme TL IM resulted in a higher degree of hydrolysis as compared to that for CRL, the amount of free LA, determined by the HPLC analysis, was similar to that for CRL. In addition, under the final optimized conditions, there was an insignificant statistical (*P* > 0.05) difference in the degree of hydrolysis among the selected lipases. On the basis of the experimental findings, CRL was selected as the most appropriate biocatalyst for the hydrolysis of SO.

### 3.7. Characterization of End Products of SO Hydrolysis by CRL

**Figure 6:**
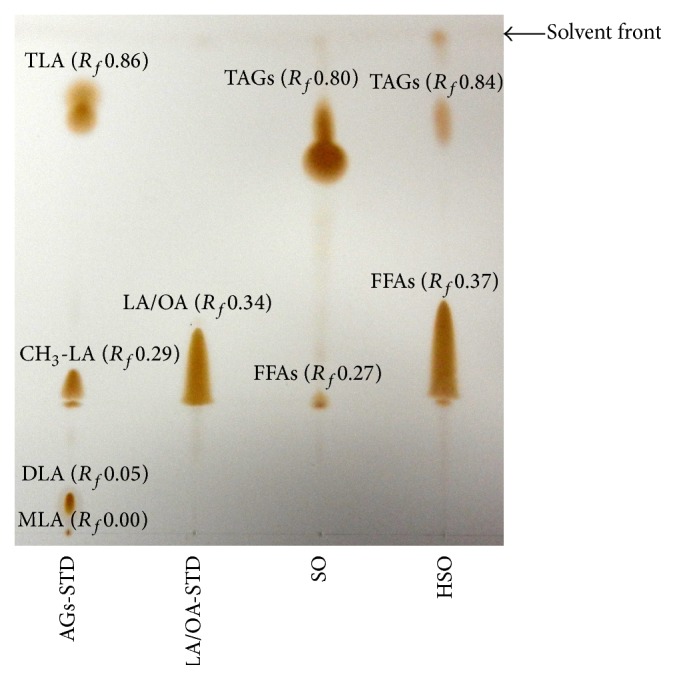
Thin-layer chromatography separation of the components of safflower oil (SO) and hydrolyzed safflower oil (HSO) by lipase from* Candida rugosa*. Acylglycerols standard (AGs-STD) was composed of 25% equal amount of methyl linoleate (CH_3_-LA), monolinolein (MLA), dilinolein (DLA), and trilinolein (TLA), whereas a mixture of free fatty acid standards (LA/OA-STD) was composed of 67% linoleic acid (LA) and 33% oleic acid (OA). Free fatty acids, FFAs, and triacylglycerols, TAGs. Retention factor (*R*_*f*_) was defined as the migration distance of a component over that of the solvent.

The SO and its hydrolyzed product (HSO) were first analyzed by TLC (Figure 6), using acylglycerols standard (AGs-STD), composed of 25% equal amount of methyl linoleate (CH_3_-LA), monolinolein (MLA), dilinolein (DLA), and trilinolein (TLA), as well as a mixture of free fatty acid standards (LA/OA-STD), composed of 67% linoleic acid (LA) and 33% oleic acid (OA); the retention factor (*R*_*f*_) was used to characterize and to identify the different components of SO and HSO. Figure 6 shows that only two bands at close positions were obtained for SO and HSO, with the first one having *R*_*f*_ value of 0.27 and 0.37 for SO and HSO, respectively, characteristic of CH_3_-LA, and the mixture of LA/OA, whereas the second band has *R*_*f*_ value of 0.80 and 0.84 for SO and HSO, respectively, characteristic of the triacylglycerols (TAGs). There was a lack in mono- (MAGs) and diacylglycerols (DAGs) presence in the HSO, which could suggest that the hydrolysis of SO resulted mainly in FFAs and trace amount of TAGs.

**Figure 7:**
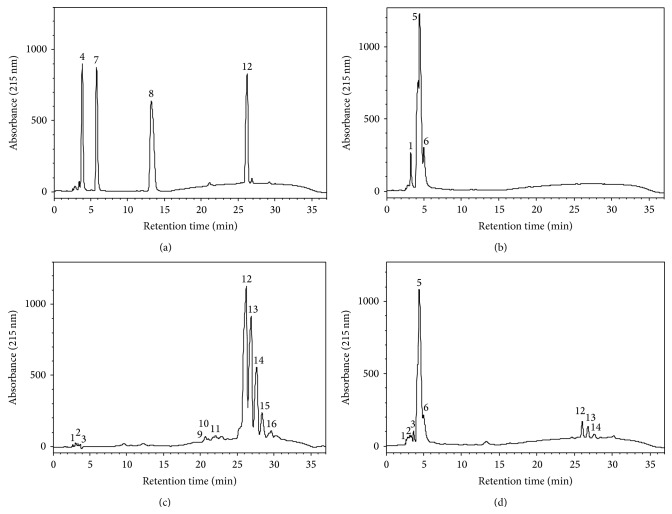
HPLC chromatograms at 215 nm of (a) acylglycerols standard (AGs-STD) composed of 25% equal amount of methyl linoleate (CH_3_-LA), monolinolein (MLA), dilinolein (DLA), and trilinolein (TLA), (b) mixture of free fatty acid standards (LA/OA-STD) composed of 67% linoleic acid (LA) and 33% oleic acid (OA), (c) safflower oil (SO), and (d) hydrolyzed safflower oil (HSO). Peak numbers were identified as follows: free fatty acids (FFAs) #1 to 3, 5, and 6, triacylglycerols (TAGs) #9 to 16, MLA #4, CH_3_-LA #7, DLA #8, and TLA #12.

Figures 7(a) and 7(b) demonstrate the elution profile of the HPLC analysis of AGs-STD and LA/OA-STD, respectively, whereas Figures 7(c) and 7(d) show that of SO and HSO, respectively. Peaks #4, 7, 8, and 12 (Figure 7(a)) correspond to MLA, CH_3_-LA, DLA, and TLA, respectively, whereas, peaks #1, 5, and 6 (Figure 7(b)) correspond to linoleic and oleic acids. The TAGs of SO were identified as peaks #9 to 16 (Figure 7(c)), with peak #12 characteristic of TLA. After hydrolysis of SO by CRL, the experimental results (Figures 7(c) and 7(d)) show a significant decrease in TAGs, with the appearance of two new major peaks, #5 and 6, which were characterized along with peaks #1, 2, and 3 as FFAs. Using a calibration curve and a linear equation of *y* = 2000000*x* + 729216, with *R* ^2^ of 0.9813, the trilinolein concentration in the HSO was determined to be 8.19 mM (data not shown). The experimental results obtained by HPLC complemented those of TLC analysis. The absence of MAGs and DAGs in the HSO could be due to the nonspecificity of CRL as opposed to the other specific lipases, such as PPL and Lipozyme TL IM [2, 23].

The relative content of FFAs in SO and HSO (Table 1), obtained by HPLC, was found to be 1.4 and 91.1%, respectively, with a net value of 89.7% for the FFAs in HSO which is close to the optimum degree of hydrolysis of SO (84.1%), determined by titration. Gas chromatography (GC) analysis of HSO was carried out to determine its relative composition in FFAs (data not shown). The composition of FFAs of HSO was determined to be C_16:0_, C_18:0_, C_18:1_, and C_18:2_, with a relative content of 8.3, 3.6, 15.3, and 72.8%, respectively. The relative FA composition of HSO was found to be close to that of SO; these experimental findings could be attributed to the nonspecificity of CRL. In contrast, Freitas et al. [2] reported that while the soybean oil has a low percentage of oleic and palmitic acids in its composition, high concentrations of these FAs were found in the CRL-hydrolyzed soybean oil.

## 4. Conclusion

The results gathered in this study showed that, among the investigated catalysts, the lipase from* Candida rugosa* was found to be the most appropriate one for the hydrolysis of safflower oil (SO); its use resulted in high concentrations of free linoleic acid (LA) that can be used as a substrate for the production of the flavor precursors, linoleic acid hydroperoxides. The overall experimental findings could contribute to the development of a bioprocess for the synthesis of natural flavor precursors by a biotechnological approach that is economically viable.

## Figures and Tables

**Table 1:** Relative content of safflower oil and its hydrolyzed product in free fatty acids, mono-, di-, and triacylglycerols, as determined by high-performance liquid chromatography (HPLC).

Component	Relative content (%)	
	Safflower oil	Hydrolyzed safflower oil
Free fatty acids^a^	1.4 (14.32)^b^	91.1 (1.12)^b^
Monoacylglycerols	n.d.^c^	n.d.^c^
Diacylglycerols	n.d.^c^	n.d.^c^
Triacylglycerols^d^	98.6 (0.21)^b^	8.9 (11.42)^b^

^a^Relative content was calculated as the total peak area of free fatty acids divided by the peak area of triacylglycerols and total free fatty acids, multiplied by 100.

^b^Relative percent standard deviation was calculated as the standard deviation of triplicate samples divided by their mean multiplied by 100.

^c^Not detected.

^d^Relative content was calculated as the peak area of triacylglycerols divided by the peak area of triacylglycerols and total free fatty acids, multiplied by 100.
